# Implications of increasing Atlantic influence for Arctic microbial community structure

**DOI:** 10.1038/s41598-020-76293-x

**Published:** 2020-11-06

**Authors:** Michael Carter-Gates, Cecilia Balestreri, Sally E. Thorpe, Finlo Cottier, Alison Baylay, Thomas S. Bibby, C. Mark Moore, Declan C. Schroeder

**Affiliations:** 1 Cellular and Molecular Department, The Marine Biological Association of the UK, Plymouth, PL1 2PB UK; 2 British Antarctic Survey, Cambridge, CB3 0ET UK; 3 Scottish Association for Marine Science, Oban, PA37 1QA Argyll UK; 4 Department of Arctic and Marine Biology, University of Tromsø - The Arctic University of Norway, 9037 Tromsø, Norway; 5 Ocean and Earth Sciences, University of Southampton, Southampton, SO14 3ZH UK; 6 Veterinary Population Medicine, The University of Minnesota, St Paul, MN 55108 USA; 7 School of Biological Sciences, University of Reading, Reading, RG6 6AH UK

**Keywords:** Water microbiology, Microbial ecology

## Abstract

Increasing influence of Atlantic water in the Arctic Ocean has the potential to significantly impact regional water temperature and salinity. Here we use a rDNA barcoding approach to reveal how microbial communities are partitioned into distinct assemblages across a gradient of Atlantic-Polar Water influence in the Norwegian Sea. Data suggest that temperate adapted bacteria may replace cold water taxa under a future scenario of increasing Atlantic influence, but the eukaryote response is more complex. Some abundant eukaryotic cold water taxa could persist, while less abundant eukaryotic taxa may be replaced by warmer adapted temperate species. Furthermore, within lineages, different taxa display evidence of increased relative abundance in reaction to favourable conditions and we observed that rare microbial taxa are sample site rather than region specific. Our findings have significant implications for the vulnerability of polar associated community assemblages, which may change, impacting the ecosystem services they provide, under predicted increases of Atlantic mixing and warming within the Arctic region.

## Introduction

Marine microbial communities underpin vital global biogeochemical cycles^1^. It is therefore critical that we understand how these communities will respond to environmental change so that we can accurately predict the susceptibility of vital ecosystem services to such change. This is particularly true for regions experiencing rapid change in the marine environment, such as the Arctic, where the impacts of climate change are amplified above the global average and significant environmental perturbations are occurring^2^. The environmental processes which underpin this amplification are complex, but are hypothesised to include changes in snow/ice cover^3^, ocean circulation^4^, cloud cover^5^, atmospheric forcing^6^ and precipitation^7^. These changes are altering environmental conditions in Arctic regions, changing regional hydrography^8^, reducing sea ice extent^9^ and increasing average water temperature regionally^10,11^. The rate of sea ice loss is being driven by a positive feedback mechanism, with the transition to a seasonal ice zone predicted sometime this century^3^. Such a mechanism implies a degree of irreversibility to the environmental change in the region.

The long-term ecological consequences of such changes remain poorly resolved. This can be partly attributed to a sparsity of, and difficulty in sampling effort which has resulted in spatially and temporally limited data^12^. Technological limitations have further hindered the exploration of polar microbial communities as historical studies have focused on microscopy and culture-dependent techniques which are limited in their ability to capture the full diversity of biological systems^13,14^. DNA sequencing methods overcome these limitations, resolving the evolutionary history and phylogenetic relationships between distinct taxa^14^, commonly by targeting highly conserved rRNA genes. Regions V4–V6 of the 16S^14^ rRNA gene for bacteria, and the 18S V9 region for eukaryotes are reported as best suited for community level phylogenetic analysis^15^.

Microbial communities are affected by their current environmental conditions^16^. Relationships between community composition and salinity^17^, water column depth^18^, latitude^19^, geographic distance^20^, water column temperature^21^ and water mass of origin^22^ have been reported, with water column temperature and salinity frequently identified as the strongest predictive factors^17,21^. Polar regions present a number of unique environmental challenges to these microbial communities. Indeed, distinct ‘ecotypes’ of cosmopolitan microbes are often reported in Arctic associated marine ecosystems including ecotypes of *Emiliania huxleyi*^23^*,* and *Fragilariopsis cylindrus*^24^. It is unclear how these microbial communities will respond to future environmental perturbations, but it is reasonable to speculate that susceptibility to environmental perturbations may vary between taxonomic groups. This assumption is supported by previous reports linking decreasing Arctic sea ice extent with increased abundance of selected bloom forming phytoplankton taxa^25^, as well as observations of significant pelagic community assemblage restructuring^26^ and poleward range shifts of some taxonomic groups^27^. The thinning of sea-ice, and an increase in the proportion of first-year ice may also favour under-ice and sea-ice associated phytoplankton blooms of taxa, including Ciliates and Haptophytes^26^, which in turn could favour bacterial taxa, such as *Formosa* and *Ulvibacter* that show positive associations to phytoplankton blooms^28^. Alternatively, enhancement of melt water driven stratification may benefit halotolerant freshwater taxa, such as certain *Synechococcus* strains^19^.

It is clear that the Arctic region is experiencing a period of environmental change which is altering the oceanic boundaries between temperate Atlantic and Polar Arctic waters^2,25^, setting up a new ‘competition’ between extant microbial communities in these regions^25^. However, the degree of vulnerability and long-term consequences of environmental change to local biological and ecosystem processes remain largely unknown, and it is unclear which community members will be “winners” or “losers” under these pressures. Therefore, key questions remain as to how microbial communities will change in response to the alteration of boundaries between Polar and temperate waters resulting from increased Atlantic Water intrusions and warming within the Arctic^29^, and whether the presence of taxa adapted to particular habitats could replace resident communities under changing environmental conditions.

We apply Illumina Next Generation Sequencing (NGS) technology to analyse microbial communities across a transect of five sampling stations in the Norwegian Sea featuring varying levels of influence from Polar Water. This allows us to directly compare the microbial community across a natural temperature and salinity gradient and assess the susceptibility of these communities to predicted alterations of these factors under increased Atlantic Water influence within the Arctic by examining community partitioning and correlations to environmental factors.

## Results

### Physical setting

**Figure 1:**
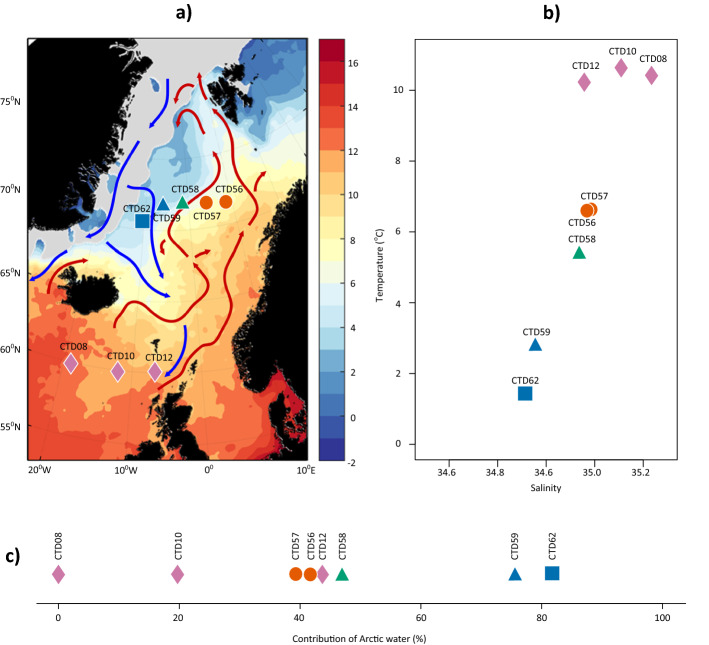
(**a**) Sea surface water temperature (SST) map with the locations of the stations sampled in the Norwegian Sea as part of UK Ocean Acidification research program during cruise JR271 (1st June 2012 to 2nd July 2012). Colour scale represents remotely sensed SST during the sampling period; grey shading indicates sea ice extent. Surface currents are illustrated by coloured arrows, blue—Polar Water currents, red—Atlantic Water currents. Symbols represent the assigned regional group determined from daily SST maps over a 6 month period prior to sampling; square—constant influence from Polar Waters, triangle—intermittent periods of Polar Water influence, circle—little Polar Water influence. Symbol colours represent the degree to which Polar Water influenced the site at time of sampling determined from in situ environmental physical characteristics measured over the sampling period; blue—most highly influenced by Polar Water, green—moderately influenced, orange—little influence. Pink diamonds show stations sampled in the North Atlantic. (**b**) Temperature/salinity plot of water properties at the deep chlorophyll maximum (10–50 m) over the sampling period. Stations separate along gradients of both temperature and salinity. Symbols and colours represent previously assigned regional groupings. (**c**) Quantification of the extent of Polar Water influence at each station, at the deep chlorophyll maximum, expressed as percentage contributions. Polar end members were defined as featuring a salinity ≤ 34.5^30^. Atlantic end members were defined as 35.4, as observed at CTD08, which featured the greatest salinity.

Daily maps of sea surface temperature (SST), and circulation data for the sampled region 6 months prior to sample collection allowed the identification of three regional groups (Fig. 1a). Images for 1 month before and 1 month after the sampling period at 2 week intervals are shown in Supplementary Information S1. The assigned regional groups reflected locations where waters were observed to experience continuous influence from Polar Water (squares), those that experienced periods of, but not constant Polar Water influence (triangles), and those that experienced little Polar Water influence (circles) during the period that SST maps were generated.

In situ measurements of the water column temperature and salinity at the deep chlorophyll maximum (Supplementary Informations S2 and S3) resolved that the sampled stations covered a natural temperature and salinity gradient indicative of differing degrees of Polar and Atlantic Water influence (Fig. 1b) during the sampled period. These measurements were used to objectively classify stations into three groups according to the extent of Polar Water influence calculated by way of a standard mixing line (Fig. 1c), Polar end members were defined as featuring a salinity ≤ 34.5 and Atlantic end members were defined as 35.4, as observed at CTD08, which featured the greatest salinity. The first group comprised stations under a high influence (blue) (76–82%), the second was of moderate influence (green) (47%) and the third of relatively low influence (orange) (39–42%) (Fig. 1a). The sample at station CTD62 (blue square) was assigned as under high influence (HI) due to SST and salinities closest to the < 0 °C and 34.5 cut offs typically used for classifying Polar Waters^30^ (Supplementary Information S3). Stations CTD56 and CTD57 (orange circles) were labelled as under low influence (LI) due to being located in a region where the SST maps showed little influence of Polar Waters and featured water temperatures > 6 °C during the sampling period. The sample taken at station CTD58 (green triangle) was observed to be in a mixed region, and featured SST and salinities between the HI and LI groups. This station was therefore labelled as under moderate influence (MI). SST maps showed that CTD59 (blue triangle) was also found in a mixed region, but in situ environmental data indicated that it was highly influenced by Polar Waters at the time of sampling due to featuring SST and salinity profiles similar to the other HI station (Fig. 1b, Supplementary Informations S2 and S3). For purposes of comparison, we also included samples collected at stations CTD08, CTD10 and CTD12 present in the North Atlantic Ocean (pink diamonds) that featured SST > 10 °C.

### Microbial community assessment

Samples were filtered through a 0.45 µm filter***. ***Sequencing of the 16S and 18S rRNA genes recovered from the stations outlined above (Fig. 1) resolved the bacterial and eukaryotic diversity at each station. Rare OTUs accounted for 86% of eukaryotic and 91% of bacterial OTUs, despite only constituting 2% and 18% of the abundance of sequences of each respective community.

Principal coordinate analysis revealed a total of 79.2% of the variance in the bacterial community, and 72.1% of the eukaryotic variance were explained by the first and second components (Supplementary Information S4). Pearson correlation analysis revealed SST as the strongest physical environmental factor that correlated with the observed variance in both communities (bacteria; *p* < 0.01, eukaryotes; *p* < 0.05), salinity was also significant for the bacterial community (*p* < 0.01) (Supplementary Information S5).

Significant correlations with other environmental factors were also observed. The bacterial community correlated with silicate, and correlations with phosphate and ammonium were observed for the eukaryotic community.

### Bacterial community

**Figure 2:**
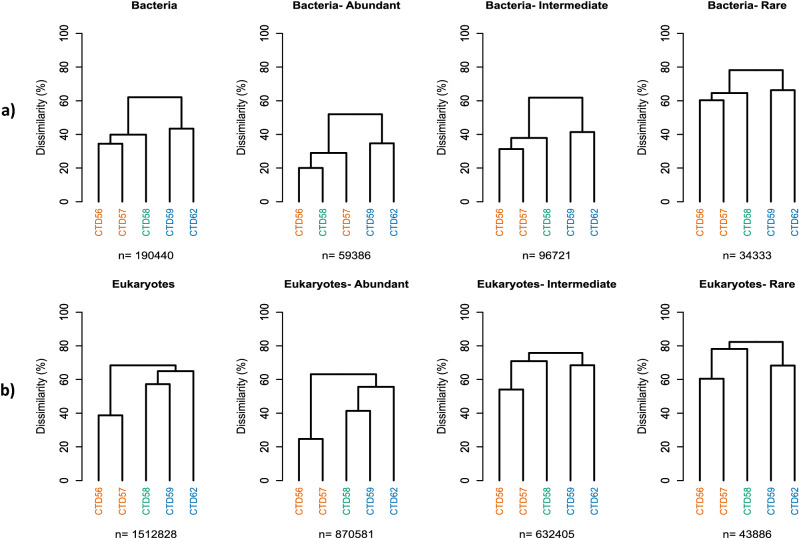
Regional partitioning of communities based upon dendrograms of the Bray–Curtis dissimilarity matrix of OTUs between stations. Shown are the (**a**) entire sampled bacterial and (**b**) eukaryotic communities, as well as each abundance fraction of the respective communities; the abundant fraction composed of OTUs representing ≥ 1% of each community, the intermediate fraction composed of OTUs representing 0.01–1% of each community, and the rare fraction composed of OTUs representing ≤ 0.01% of the entire community. Stations are coloured based upon the extent of Polar Water influence determined to be present at each station as in Fig. 1; orange-LI, green-MI, blue-HI.

The bacterial (16S) community was composed of 10,272 distinct OTUs (Supplementary Information S6). The bacterial community partitioned into two distinct clusters (dissimilarity 65%) that mirrored the regional assignment of stations based on the physical data, with the two LI stations (CTD56 and CTD57) in one cluster, and two HI stations (CTD59 and CTD62) in the other (Fig. 2a). The sample collected at station CTD58, the MI sample, was more dissimilar to the samples collected at the HI stations. This partitioning was repeated at all abundance fractions of the community (Fig. 2a).

**Figure 3:**
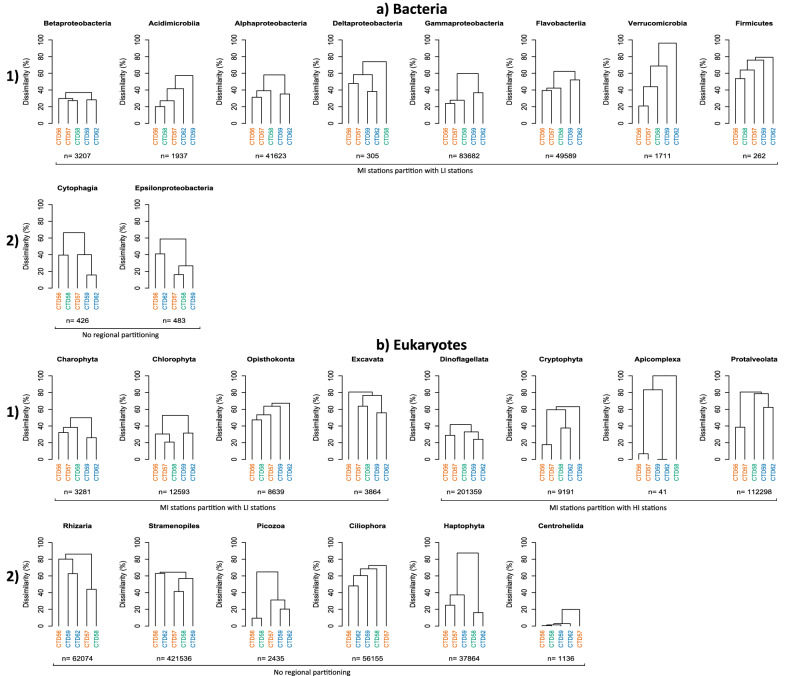
Regional partitioning of communities based upon dendrograms of the Bray–Curtis dissimilarity matrix between stations for (**a**) the entire bacterial and (**b**) entire eukaryotic community divided into representative major taxonomic groups. (1) Shown are those groups which display regional partitioning which matches the regional assignment of stations from SST map analysis. Constituent bacterial taxonomic groups displayed partitioning for which the MI station was most dissimilar to the HI stations. Eukaryotic taxonomic groups displayed contrasting partitioning and are separated into those for which the MI station was most dissimilar to the HI stations, and those where the MI station was most dissimilar to the LI stations. Taxonomic groups are ordered by dissimilarity of the MI station to the LI or HI stations from least to most dissimilar (left to right). (2) Also shown are those taxonomic groups which displayed no clear regional partitioning. Stations are coloured based upon the extent of Polar Water influence determined to be present at each station as in Fig. 1; orange-LI, green-MI, blue-HI. The number of sequences comprising each taxonomic group is shown (n).

The defined bacterial OTUs could be assigned to 10 main taxonomic groups (Supplementary Information S7). These ten groups represented 96.2% of the total bacterial community abundance. Gammaproteobacteria was the most abundant group at all stations, followed by Flavobacteriia and Alphaproteobacteria. Eight of the 10 taxa, namely the Alpha-, Beta-, Delta-, Gamma-proteobacteria, Acidimicrobiia, Flavobacteriia, Verrucomicrobia and Firmicutes, all displayed strong regional partitioning (dissimilarity of 40–80% between LI and HI station groups) (Fig. 3a.1). The taxonomic composition within seven of these eight taxonomic groups at the MI station was more dissimilar to that of the HI stations. The notable exception being the MI Deltaproteobacteria where the composition was 75% dissimilar to that found in both LI and HI stations. Two taxa, namely the Epsilonproteobacteria and Cytophagia (Fig. 3a.2) showed no regional partitioning. Based on this analysis we infer that 7 bacterial taxonomic groups show regional clustering where MI stations were more dissimilar to HI stations.

**Figure 4:**
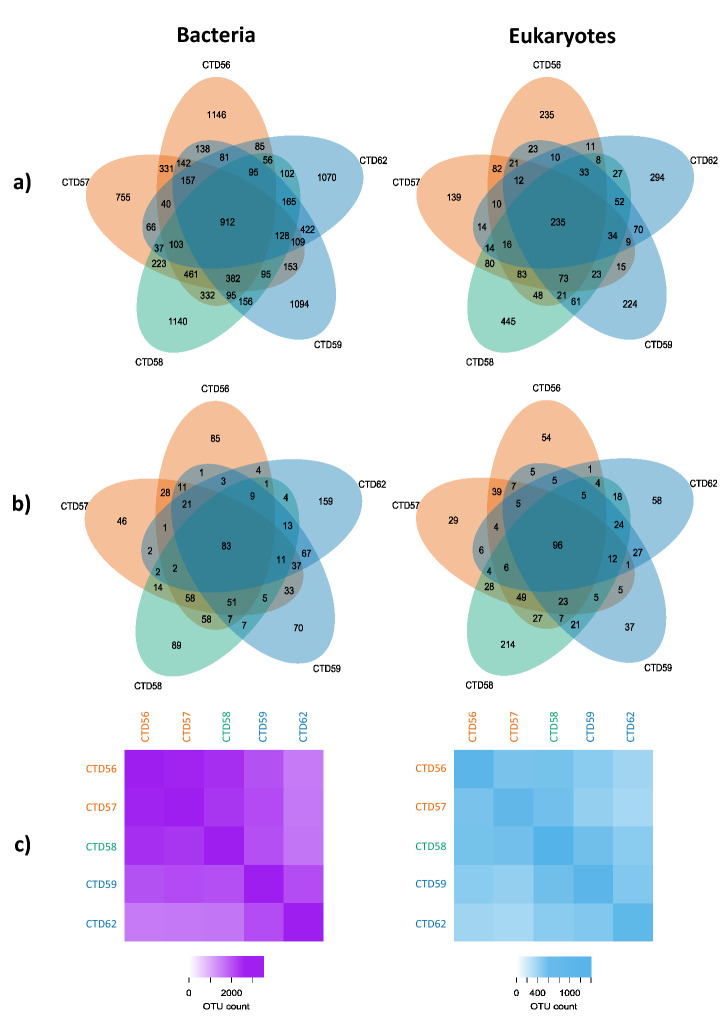
Analysis of shared and unique bacterial and eukaryotic OTUs found within stations across the transect. Shown are the OTUs shared between stations, and those unique to stations for (**a**) the entire sampled community, as well as (**b**) with those OTUs represented by < 10 sequences at any one station excluded for both the bacterial (left) and eukaryotic communities (right). Numbers represent the count of unique OTUs. OTU counts in station ellipses which do not overlap with any other represent the number of unique OTUs specific to that station, whereas those that do overlap represent the number of OTUs found within those stations for which ellipses overlap. Also shown is (**c**) the number of unique OTUs shared between each pair of stations, with the number of OTUs represented by the colour scale, lighter shades indicate fewer OTUs. Stations are coloured according to the degree of Polar Water influence as in Fig. 1; orange-LI, green-MI, blue-HI.

Further exploration of the bacterial OTUs revealed that each station had a number of unique OTUs that could only be found at that individual station (Fig. 4a). When OTUs represented by < 10 sequence copies per OTU were excluded (Fig. 4b) a similar pattern remained. Thus, the number of unique OTUs found at each station were not skewed by artefacts from OTUs with low sequence number. A pool of “core OTUs” were common to all stations, 912 in total for bacteria (Fig. 4a). Bacterial HI stations were observed to share fewer OTUs with the MI and LI stations, indicating compositional differences between the station groups (Fig. 4c).

**Figure 5:**
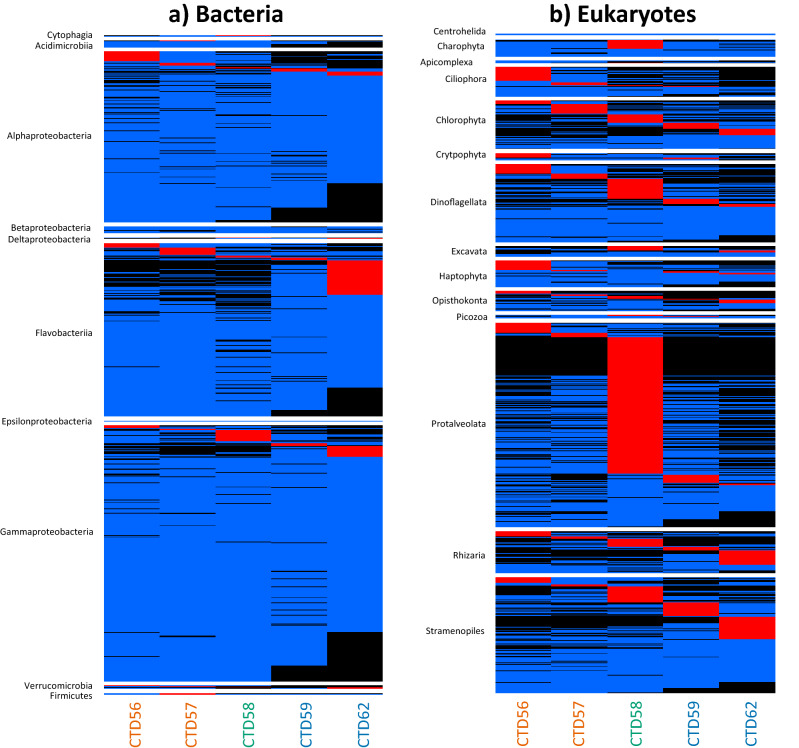
Heatmaps of the proportional contribution each OTU makes to the community across all stations for (**a**) the bacterial, and (**b**) the eukaryotic community, divided into constituent taxonomic groups. Shown is the proportional contribution each OTU makes to the community across all stations. Black represents absent OTUs that make no contribution to the community at a particular station, blue represents > 0–60%, and red represents ≥ 60% at one station. A T10 cut off was used to reduce artefacts resulting from low sequence number. Stations are coloured according to the degree of Polar Water influence as in Fig. 1; orange-LI, green-MI, blue-HI.

Heatmaps resolved 391 OTUs that displayed a strong proportional bias to certain stations indicating preferential conditions at individual stations. The Flavobacteriia featured 151 OTUs that were deemed to display a strong proportional bias (red) to a particular station, 101 of which were identified at the HI station CTD62 (Fig. 5a). An absence (black) of these OTUs at CTD56, CTD57 and CTD58 was also observed. The Alphaproteobacteria, Acidimicrobia and Gammaproteobacteria featured 71, 3 and 94 OTUs, respectively, which displayed a proportional bias to a particular station. Of these OTUs the highest numbers were identified at CTD56 (32), CTD57 (2) and CTD58 (37) for the Alphaproteobacteria, Acidimicrobia and Gammaproteobacteria respectively. An absence of OTUs at HI stations was again observed for these three taxonomic groups. No representatives of Betaproteobacteria were deemed to show strong proportional bias. All other taxonomic groups featured few OTUs making it difficult to draw conclusions likely to be reflective of the group.

### Eukaryotic community

The eukaryotic (18S) community was composed of 2558 distinct OTUs (Supplementary Information S6), and also displayed regional partitioning into two distinct clusters (dissimilarity of 70%) (Fig. 2b). However, contrasting partitioning was observed whereby the MI station (CTD58) clustered with the HI stations (CTD59 and CTD62) for the entire sampled community and abundant fraction of the filtered communities, but the MI station partitioned with the LI stations for the rare and intermediate abundance fractions (Fig. 2b). Identical regional partitioning was observed for both bacterial and eukaryote communities when samples collected from the North Atlantic were included in the analysis, providing a further comparison against a temperate community (Supplementary Information S8).

Compositional differences within the eukaryotes included the HI stations being dominated by Stramenopiles, whereas the LI stations were dominated by a high relative abundance of an uncultured SAR (Alveolate) isolate NIF-4C10, and Dinoflagellates (Supplementary Information S7). The MI station featured elevated levels of Proteoalveolata compared to other stations.

Analysis of the constituent eukaryotic taxonomic groups revealed that eight; namely the Charophyta, Chlorophyta, Dinoflagellata, Excavata, Protalveolata, Apicomplexa, Opisthokonta and Cryptophyta displayed strong regional partitioning (dissimilarity of 50–85% between the HI and LI station groups) (Fig. 3b.1). The taxonomic composition within four of these taxonomic groups, the Charophyta, Chlorophyta, Excavata and Opisthokonta found at the MI station was most dissimilar to those at HI stations. For the Dinoflagellata, Protalveolata and Cryptophyta the MI station was most dissimilar to LI stations. The MI station CTD58 was highly dissimilar to both the LI and HI stations for the Apicomplexa taxon. Despite observed differences in relative abundance (Supplementary Information S7), the Rhizaria, Stramenopiles, Picozoa, Ciliophora, Haptophyta and Centrohelida (Fig. 3b.2) displayed no clear regional partitioning. Analysis was not possible for the Rhodophyta or Ameobozoa due to insufficient recovery of representative OTUs at individual stations.

Heatmaps of eukaryotic OTUs displaying a strong proportional bias to certain stations (red) revealed they were more common than observed in the bacterial community (604 eukaryotic OTUs vs 391 bacterial OTUs). 189 of the 223 Protalveolata OTUs featuring a strong proportional bias was observed at CTD58, with a number absent at all other stations (black) (Fig. 5b). Within the Charophyta and Excavata most OTUs displaying a strong proportional bias was observed at CTD58, being 12 and 6, respectively. The Chlorophyta, Haptophyta and Cryptophyta displayed the most biased OTUs at CTD56, being 21, 13 and 6, respectively. The Rhizaria featured 20, and the Stramenopiles 31, biased OTUs at CTD62. Representatives of the Ciliophora, Opisthokonta and Dinoflagellata featured a similar number of station biased OTUs across all stations.

## Discussion

This study explored the microbial communities present across an oceanographic gradient of Atlantic-Polar Water influence in the Norwegian Sea and revealed how microbial communities are being partitioned into distinct assemblages. Communities were observed to display specific distribution patterns that correlated with defined physical oceanographic characteristics, as is supported by studies that have reported geographically distinct polar communities^31,32^, and large scale studies that have reported temperature as a main predictive variable of global epipelagic microbial community structure^21^, although these exclude the Arctic region.

The reason for observed correlations of the bacterial community with silicate is unclear, but representatives of Flavobacteriia and Gammaproteobacteria are known to associate with diatoms^33^ which may offer an explanation. Phosphate is often suggested to be a growth limiting nutrient within freshwater phytoplankton communities^34^, but phosphate limitation of marine communities has also been demonstrated^35^. Nitrogen is typically considered to be the main growth limiting nutrient of marine phytoplankton^36^, which may be preferentially uptaken in the form of ammonium rather than nitrate^37^, therefore, the observed correlations of the eukaryotic community with phosphate and nitrogen are largely expected.

The partitioning of the MI station community, present in a mixed region, in relation to the LI and HI communities provides a potential means to predict which community may become dominant under increased Atlantic Water influence within the region. The observed correlations with environmental variables and partitioning of the MI station with the LI station for the bacterial communities implies that cold water bacterial OTUs found at highly Polar Water influenced regions could potentially be displaced under predicted increased Atlantic mixing and warming within the Arctic region. A similar trend is also suggested for OTUs within the rare and intermediate abundance fractions of the eukaryotic community. However, the MI station partitioning with HI stations for the abundant eukaryotic community fraction, suggests that OTUs representative of highly Polar Water influenced regions could dominate over warmer adapted temperate where these communities mix. Analysis of the major taxonomic groups within each community showed that partitioning which matched the regional assignment of stations based on environmental data was a common feature. However, not all groups partitioned in the same way, implying that intra-taxa responses to environmental pressures are more complex. Data for the bacterial taxa Alpha-, Beta-, Gamma-proteobacteria, Acidimicrobiia, Flavobacteriia, Verrucomicrobia and Firmicutes revealed that there is the potential for community restructuring to result in a displacement of cold water associated OTUs. A similar potential exists for eukaryotes within the taxonomic groups Charophyta, Chlorophyta, Excavata and Opisthokonta. However, certain members of the Dinoflagellata, Protalveolata and Cryptophyta may undergo a contrasting restructuring, whereby constituent cold water OTUs may replace warmer adapted temperate OTUs under conditions where populations mix.

As is common in NGS studies, the majority of OTUs were observed to belong to rare members of the community, here defined as those representing ≤ 0.01% of the community. It is this rare component of the microbial community that represents the vast majority of the biological diversity in the oceans^38^. Little is known about the ecological or functional role of members of the rare community, but there is evidence that some rare species can display high levels of metabolic activity enabling them to disproportionally contribute to certain ecosystem functions^39^. The rare community may also play a key role in the resilience of an ecosystem through acting as a seedbank of a large number of functionally redundant similar genetic variants, adapted to slightly different environmental conditions. As environmental perturbations occur, the abundance of these variants changes with little overall effect on ecosystem function^40^. Indeed, compensatory reactions of rare phytoplankton taxa to environmental stressors have been evidenced to maintain core ecosystem functions under experimental conditions^41^. Yet the window of tolerance for these different variants is likely to be small and unable to cope with larger environmental changes^42^, and are suggested as the most vulnerable taxa to local extinctions^43^. The partitioning of the rare fraction of both the bacterial and eukaryotic community implied the likely displacement of cold water associated OTUs with Atlantic associated OTUs under increased Atlantic Water influence, therefore, local loss of cold adapted rare taxa may have implications to the regions resilience to future change.

A core pool of OTUs was recovered from all stations, implying ecological viability throughout a range of environmental conditions. Despite these OTUs being present at all stations, examples were observed that were proportionally more abundant at certain stations, and less so elsewhere (Supplementary Informations S9 and S10). This suggests a growth response in reaction to favourable conditions found at individual stations and raises questions over the impacts of deviations from these conditions for such OTUs. OTUs displaying such abundance profiles were common within the eukaryotic community, but rarer within the bacterial community. However, visual trends of the abundance profiles of bacterial OTUs show a more graduated abundance pattern across stations, which may in part be driven by the greater ease of bacterial dispersal over regional distances compared to larger eukaryotes^44^. Additionally, it may be that greater taxonomic resolution may be required to resolve stronger ecological patterns within the bacterial community that are missed even at the OTU level^45^.

In addition to predicted changes to the community as a whole, and constituent taxonomic groups, certain individual OTUs may also be particularly susceptible to changing conditions. Heat map data showed the Chlorophyta, Cryptophyta and Epsilonproteobacteria featured OTUs that displayed a strong proportional bias towards CTD56, implying a preference of these individuals for LI waters. As the alterations to the boundaries between Atlantic and Polar Waters^2^, as well as warming of the Arctic region continues, such taxa will likely experience positive selection, increasing in abundance and extending their geographic range, as already seen for other example taxa^23,27,46^. By contrast, the Verrucomicrobia, Rhizaria, Flavobacteria and Stramenopiles featured high number of OTUs found in greater proportions at CTD62, which are potentially susceptible to local exclusion as suitable habitat range contracts. Similar examples were observed in nearly every taxonomic group, and at multiple stations, implying the presence of individuals within all taxonomic groups and at all stations that are at risk to environmental perturbations.

Our findings of community members potentially susceptible to increased Atlantic influence are validated by known patterns in the current literature. For example, the Flavobacteriia genus *Polaribacter* contained potentially temperature-dependent ecotypes, agreeing with previous reports of psychrophilic^47^ or cold water restricted^22^ members. HI stations also featured elevated levels of *Ulvibacter* (also a Flavobacteriia), which were first isolated from a polar environment^48^. Similar patterns were seen for the Gammaproteobacteria genus *Balneatrix*, which were found at high abundances at the HI station, and were originally isolated from freshwater^49^. A number of taxa were recovered at LI and MI station groups that are known to display temperature driven distributions and are typically associated with temperate waters. These included the SAR86 clade^50^, ZD0405 clade^51^ of the Gammaproteobacteria, and Rhodobacteraceae^52^, as well as SAR11 subclades^53^. Due to the primer used, SAR11 diversity may be under estimated^54^. Within the eukaryotes there was a clear difference within the Haptophytes which mirrored previous reports, the LI stations were primarily occupied by representatives of *Coccolithales* which were replaced with *Phaeocystis* at the HI stations, a taxon known to contain members that associate with cold regions^55^.

Here we have demonstrated that the pelagic microbial community present across an oceanographic gradient of Polar Water influence across the Norwegian Sea displays specific distribution patterns that correlated with hydrography. The distinct genetic distance observed between the taxa found within HI and LI groups raises important questions concerning the structuring of microbial communities under predicted future increasing Atlantic influence and warming in the Arctic region. The observed partitioning of communities suggests a likely displacement of bacterial communities found in highly Polar Water influenced areas as Atlantic Water influence increases within the region, but with the responses of eukaryotes being more complex and differing between constituent taxonomic groups. We have also shown the likely susceptibility of certain OTUs to significant changes in local abundance due to distinct abundance patterns of these individual OTUs, presumably reflecting more favourable growth conditions at specific stations. Future monitoring and assessment of the region is imperative to track and quantify the extent of these impacts if we are to determine their long-term ecological effects.

## Methods

The water samples used in this study were collected from CTD casts taken as part of cruises for the UK Ocean Acidification research program aboard the RRS James Clark Ross research vessel during cruise JR271^56^ (1st June 2012 to 2nd July 2012; for cast locations see Supplementary Information S12). This program aimed to reduce uncertainties in the predictions of changing ocean carbonate chemistry and the response of marine organisms to such stressors. The cruise comprised a transect of five stations in the Norwegian Sea (Fig. 1). Three additional stations located in the North Atlantic Ocean were also sampled.

### Sea surface temperature and circulation maps

Daily maps of absolute dynamic topography and sea surface temperature were created for the 6 month period prior to sampling for the study region. These were used to examine the mesoscale circulation of the region during sampling. High resolution (0.05°) sea surface temperature and sea ice fraction data were obtained from the Operational Sea surface Temperature and Ice Analysis (OSTIA) system using both in situ and satellite data^57^.

### CTD casts

CTD data were collected at each sampling station using a standard Rosette unit using either a stainless steel or titanium frame and equipped with the following sensors; SeaBird (St. Bellevue, Washington, USA) Digiquartz temperature compensated pressure sensor, SeaBird-SBE 4C, SBE 3P, SBE 43, Chelsea (Surrey, UK) MKIII Aquatracka fluorometer, WETLabs (Philomath, Oregon, USA) C-Star 25 cm path transmissometer, Biospherical (San Diego, California, USA) QCD-905L PAR irradiance sensor, Tritech (Westhill, Scotland) PA200 altimeter. These were used to determine the dissolved oxygen content (DO_2_) [µmol l^−1^], photosynthetically available radiation (PAR) [µmol photons/m^2^ s^−1^], pressure [dbar], density anomaly [kg m^−3^], temperature [°C], salinity and chlorophyll fluorescence [mg m^−3^] directly on site at one metre intervals spanning from just below the sea surface to the sea floor. Nitrate, ammonium and phosphate measurements were obtained by running samples through a Skalar (Breda, Netherlands) San + Segmented Flow Autoanalyzer using colourimetric techniques^58^.

### DNA extractions

Water samples were collected from each station at the deep chlorophyll maximum (Supplementary Information S3) in Nalgene bottles washed with 1.5% HCl solution and rinsed three times with MilliQ water. From each bottle 0.25–1.00 L of seawater was filtered by vacuum pump through a 0.45 µm polycarbonate membrane filter (PALL Corporation, Michigan, USA); a protocol confirmed to be sufficiently sensitive for capturing both the pro- and eukaryotic communites^59,60^. This 0.45 μm cutoff was chosen over the more traditional 0.22 μm because of the increased bias towards capturing giant marine viruses, known to encode many pro- and eukaryotic genes. We accepted that we might not efficiently capture the smaller bacterial species on the 0.45 μm filter but as stated above, we found that the microbial diversity observed was comparable to other studies using the 0.22 μm filters. This was likely because accuracy in size fractionation lies in the filtrate and not the retentate. The retentate, i.e. the filters themselves, do still capture the smaller particles. Each filter was rinsed in a petri dish with 2 ml of phosphate buffered saline (PBS) solution and the resultant solution transferred to an Eppendorf tube. DNA was extracted from the PBS solution for each environmental sample using Qiagen DNeasy Blood and Tissue kit as per the manufacturers protocol (QIAGEN, Valencia, CA, USA) before being frozen at − 20 °C for later laboratory analysis.

### Probe assay

For each sample, amplification of the V4 region of the 16S SSU rRNA gene to target the bacterial community, and V9 region of the 18S SSU rRNA gene to target the eukaryotic community was carried out in triplicate using universal primers (Supplementary Information S13) to generate DNA barcodes for taxonomic analysis. The amplified 16S V4 region spanned ~ 350 bp, and the amplified 18S V9 region spanned ~ 270 bp. 1 µl of the extracted environmental DNA was added to 5 µl Colourless GoTaq Flexi Buffer, 1.5 µl MgCl^2^ 25 mM, 2.5 µl PCR Nucleotide mix 10 mM, 1 µl Evagreen dye, 0.1 µl GoTaq DNA polymerase, 12.9 µl molecular grade water and 0.5 µl of both forward and reverse primers (10 pmol/µl) up to a final volume of 25 µl for each sample. 1 µl molecular water was used in place of extracted DNA to act as negative controls for each primer combination.

Real-time qPCR was run on a Corbette Rotor-Gene 6000 using an initial denaturation step of 94 °C for three minutes, followed by up to 35 cycles of a three step qPCR: 94 °C for 45 s, 50 °C for 60 s and 72 °C for 90 s. Each sample and its corresponding negative control were removed after the cycle in which it exceeded a fluorescence threshold of 80 to minimise the formation of artefacts such as chimeras during the plateau phase of the reaction.

### Electrophoresis gel

To confirm the success of the amplification each PCR product was loaded into a 1.5% agarose gel and run for 50 min at 110 V to separate the amplicons by size fraction and check for contamination. Bands of ~ 270 bp for the 18S samples and ~ 350 bp for the 16S samples were removed by razor blade under a UV transilluminator.

### DNA recovery

DNA was recovered from the excised gel bands for each sample using the Zymoclean Gel DNA recovery protocol as per the manufacturer’s instructions (Zymo Research, Irvine, CA, USA). The quality and quantity of recovered DNA was assessed using an Agilent DNA 1000 kit and corresponding dsDNA 12000 Series II assay (Agilent Technologies, Santa Clara, CA, USA) (Supplementary Information S14). Samples were diluted to a final concentration of 4 nM L^−1^ for each sample and the highest quality replicate for each site selected. The quality assignment of each replicate was based upon the strength of peaks generated during the Agilent assay and the total quantity of DNA recovered. Where similar quantities of DNA were recovered from two replicates the one with the narrowest peak observed during the Agilent assay was selected. 3 µl of the 4 nM L^−1^ solution of each of the chosen replicates was combined and sent for sequencing by Illumina MiSeq technology at The National Oceanography Centre in Southampton, England.

### Bioinformatic analysis

The processing of the Raw Illumina MiSeq sequences was carried out on the Biolinux platform^61^. Raw sequence quality was first assessed using FAST-QC^62^. Any over represented or primer sequences were removed using Cutadapt v1.9.1^63^. Both forward and reverse sequences were quality filtered using PEAR v0.9.8^64^ to retain only high quality sequences above a Phred score of 28, while simultaneously merging them. Sequences outside of 100–300 nucleotides long were removed. Finally, all 18S sequences were trimmed to a maximum length of 270 bp, and all 16S sequences to 250 bp using R v3.3.0^65^ to aid with alignment. These steps ensured poor quality data did not interfere with downstream processing.

For the 18S sequences, Swarm v2.1.6^66^ was used to create a single dereplicated file for the entire study which contained only unique sequences, from this an amplicon contingency table of all unique OTUs in all samples was generated. OTUs for each sample were then assigned using the Swarm v2.1.6^66^ clustering algorithm with one ambiguous nucleotide allowed between OTUs. For the 16S sequences, Qiime was used to first create a mapping file to enable all sequences from all stations to be combined into a single fasta file. All sequences were then clustered into OTUs based on a 98.7% similarity, which is suggested as most suited to resolve OTUs at the equivalent of species level^67^. Representative sequences for each OTU were selected based on the most abundant sequence for each OTU. Taxonomy was determined for each OTU for both 16S and 18S datasets using Qiime v1.9.1^68^ by blasting against the SILVA^69^ database (release 128) with an e-value threshold of 10^–8^. R^65^ was used to add the taxonomic annotation to each corresponding OTU, resulting in an output of annotated unique OTUs present in each sample and their abundances. Any singletons, defined as OTUs identified by only one sequence across the entire study were excluded. The taxonomic assignment for each OTU was manually validated against the NCBI database and amended as necessary. Each dataset was rarefied by subsampling to the smallest number of sequences recovered at a single station to normalise the data and enable comparability across stations. This was achieved by using the *“*rarefy*”* function in the R package ‘*vegan’*^70^.

### Estimations of diversity

Analysis of the sequencing effort was achieved by constructing rarefaction curves of the observed OTU richness, and extrapolated OTU richness from the rarefied dataset using the R “iNEXT” package^71^ (see Supplementary Information S15).

To analyse the diversity recovered at each station α-diversity metrics calculated as part of the rarefaction analysis using the “iNEXT” R package were recorded, and ACE diversity estimator calculated using the “estimateR” function in the R package ‘*EpiEstim’*^72^*.* A comparison of the β-diversity across the stations was achieved by generating a dendrogram of the Bray–Curtis dissimilarity matrix between OTUs recovered at each station using the “vegdist” function in the R package ‘*vegan’*^70^. This was also carried out for OTUs separated into the major taxonomic groups. The structuring of the eukaryotic community was influenced by a number of copepod OTUs, likely the result of debris rather than intact whole organisms. Removal of these resulted in reduced genetic dissimilarity of CTD59 and CTD62 (from 73 to 67%) (Supplementary Information S8), as would be expected based on the similarity of environmental characteristics (Supplementary Information S3), hence the copepods were excluded from further analysis.

Venn diagrams were created using the *‘VennDiagram’* function in the R package *‘vegan’*^70^ to visualise the distribution of shared and specific taxa by comparing the presence/absence of OTUs. The relative abundance across stations of the top 200 OTUs were also plotted using R^65^ to explore how their distribution pattern changed and explain the community structure observed. Correlations of these OTUs with environmental factors (Supplementary Informations S9, S10 and S11) were determined by Spearman’s rank analysis using the *‘rcoor’* function from the R package ‘*vegan’*^70^.

Heatmaps were generated for both the bacterial and eukaryotic community, and subdivided by their respective constituent major taxonomic groups using the R package ‘*pheatmap’*^73^. The heatmaps visualise the relative proportional contribution each OTU makes to the community across all stations^74,75^. To exclude any potential bias introduced through low sequence number any OTUs represented by < 10 reads were removed. OTUs were deemed to display a strong proportional bias for a station where the proportion of total sequences from all stations for an OTU was ≥ 60% at one station.

## Supplementary information

Supplementary Information.
