# Supplementary material for: Implications of increasing Atlantic influence for Arctic microbial community structure
Source: Sci Rep. 2020 Nov 6;10:19262. doi: 10.1038/s41598-020-76293-x (PMC7648788; doi:10.1038/s41598-020-76293-x)

**Supplementary information for:**

**Implications of increasing Atlantic influence for Arctic microbial community structure**

Authors: Michael Carter-Gates^1^, Cecilia Balestreri^1^, Sally E. Thorpe^2^, Finlo Cottier^3,4^, Alison Baylay^5^, Thomas S. Bibby^5^, C. Mark Moore^5^ and Declan C. Schroeder*^1, 6, 7^

^1^ Cellular and Molecular Department, The Marine Biological Association of the UK, Plymouth, UK, PL1 2PB

^2^ British Antarctic Survey, Cambridge, UK, CB3 0ET

^3^ Scottish Association for Marine Science, Oban, Argyll, UK, PA37 1QA

^4^ Department of Arctic and Marine Biology, University of Tromsø - The Arctic University of Norway, 9037, Tromsø, Norway

^5^ Ocean and Earth Sciences, University of Southampton, Southampton, UK, SO14 3ZH

^6^ Veterinary Population Medicine, The University of Minnesota, St Paul, MN 55108

^7^ School of Biological Sciences, University of Reading, Reading, RG6 6AH

*: corresponding author [dcschroe@umn.edu](mailto:dcschroe@umn.edu)

**S1- Sea surface temperature (SST) maps with the locations of the stations sampled in the Norwegian Sea as part of UK Ocean Acidification research program during cruise JR271 (1^st^ June 2012 to 2^nd^ July 2012) for the 2 month period before and 2 month period after sampling at 2 week intervals.** Colour scale represents measured SST during the sampling period; grey shading indicates sea ice extent. Symbols represent the assigned regional group determined from daily SST maps over a 6 month period prior to sampling; square – constant influence from Polar Waters, triangle – intermittent periods of Polar Water influence, circle – little Polar Water influence. Symbol colours represent the degree to which Polar Water influences the site determined from in situ environmental physical characteristics measured over the sampling period; blue – most highly influenced by Polar Water, green- moderately influenced, orange- little influence.


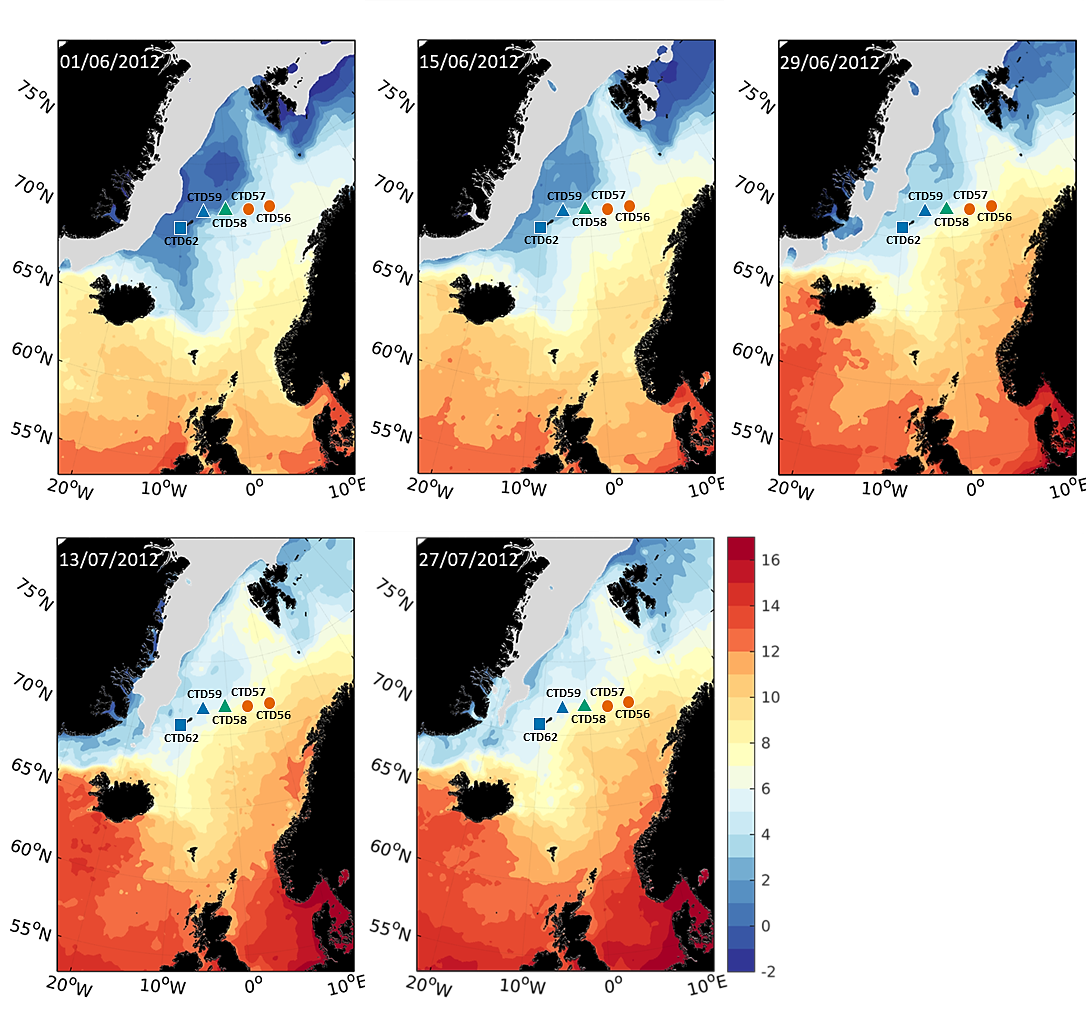


**S2- Vertical profiles of the environmental conditions at each station measured during CTD casts.** Shown are profiles for a) water temperature, b) dissolved oxygen, c) subsurface PAR, d) density anomaly, e) salinity and f) chlorophyll fluorescence. Black X donates the sampled depth at the DCM for each station.


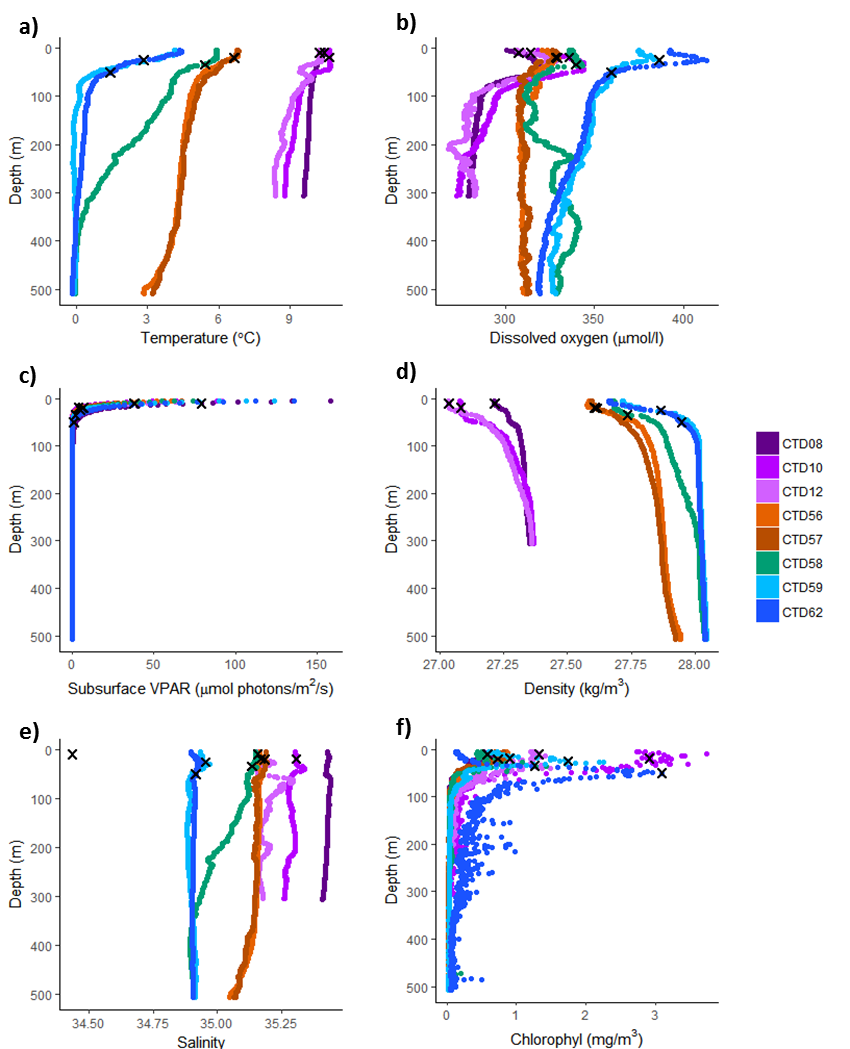


**S3-** The environmental conditions at the sampled depth for each CTD cast.

| **Station** | **Sampled depth [m]** | **Density anomaly [kg m^-3^]** | **Dissolved O2 [µmol l^-1^]** | **Salinity** | **Subsurface PAR [µmol photons /m^2^s^-1^]** | **Temperature [ᵒC]** | **Ammonium**  **(nM)** | **Nitrate (nM)** | **Silicate (nM)** | **Phosphate (nM)** |
| --- | --- | --- | --- | --- | --- | --- | --- | --- | --- | --- |
| **CTD 08** | 10.01 | 27.215 | 307.4 | 35.434 | 79.11 | 10.437 | 931.50 | 6.53 | 4.27 | 0.45 |
| **CTD 10** | 19.02 | 27.082 | 335.7 | 35.309 | 3.79 | 10.635 | 159.84 | 2.94 | 1.45 | 0.21 |
| **CTD 12** | 10.01 | 27.036 | 314.3 | 35.157 | 37.97 | 10.225 | 69.78 | 6.06 | 1.68 | 0.40 |
| **CTD 56** | 19.00 | 27.606 | 328.2 | 35.169 | 6.56 | 6.613 | 499.20 | 5.54 | 5.09 | 0.43 |
| **CTD 57** | 20.00 | 27.614 | 329.0 | 35.185 | 5.87 | 6.644 | 284.86 | 5.92 | 4.57 | 0.42 |
| **CTD 58** | 34.01 | 27.735 | 339.5 | 35.136 | 1.82 | 5.407 | 1036.47 | 8.97 | 5.27 | 0.68 |
| **CTD 59** | 25.01 | 27.866 | 386.2 | 34.955 | 5.11 | 2.817 | 1101.83 | 7.19 | 2.27 | 0.59 |
| **CTD 62** | 50.02 | 27.948 | 359.5 | 34.916 | 0.19 | 1.430 | 1204.59 | 7.93 | 3.16 | 0.64 |

**S4- Principle coordinate analysis of the a) bacterial and b) eukaryotic community.** 79.2% of the variance of the bacterial community was explained by the first and second principle components (PC), with 63.6% explained by PC1 and 15.6% by PC2. For the eukaryotic community 72.1% of the variance in the eukaryotic community was explained, with 45.2% explained by PC1 and 26.9% by PC2. Stations are coloured based upon the extent of Polar Water influence determined to be present at each station as in Figure 1; orange- LI, green- MI, blue- HI.

**
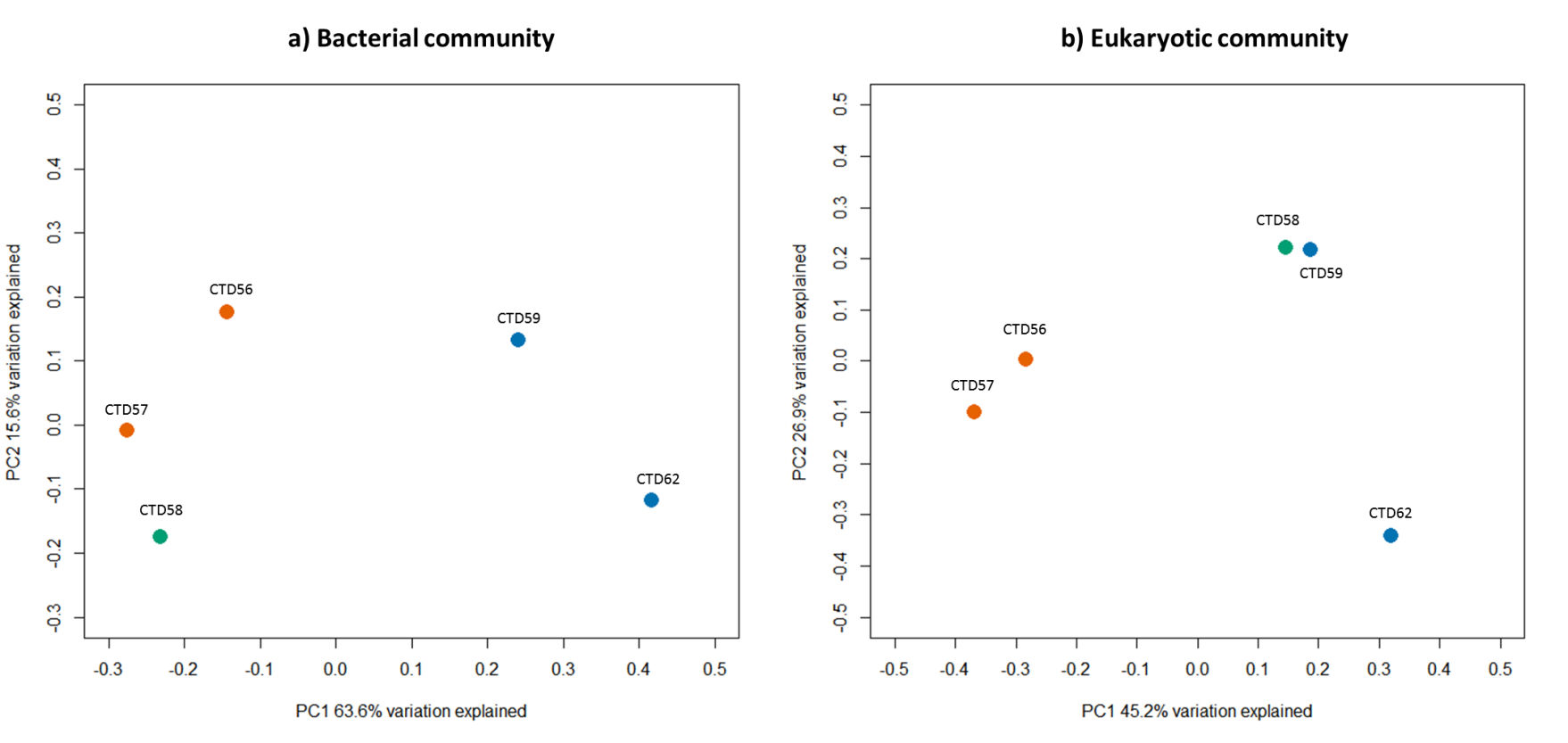
**

**S5- Pearson correlation of environmental metadata to community variance.** * donates *p*<0.05. ** donates *p*<0.01. For the bacterial community Pearson correlation of environmental data with PC1 and PC2 revealed temperature and salinity as the strongest correlating factors (*p*<0.01), silicate was also significant (*p*<0.05). No factors significantly correlated with PC2. For the eukaryotic community temperature was revealed as the greatest explanatory physical environmental factor (*p*<0.05). Ammonium and phosphate were the strongest nutrient factors (*p*<0.05). No factors significantly correlated with PC2.

|  |  | **Eukaryotes** | |  |  | **Bacteria** |  |  |
| --- | --- | --- | --- | --- | --- | --- | --- | --- |
|  |  | **PC1** |  | **PC2** |  | **PC1** |  | **PC2** |
|  | **R** | **P** | **R** | **P** | **R** | **P** | **R** | **P** |
| **Dissolved O_2_** | 0.74 | 0.15 | 0.19 | 0.76 | 0.79 | 0.11 | 0.13 | 0.83 |
| **Salinity** | -0.84 | 0.08 | 0.27 | 0.66 | -0.96 | **0.01 | 0.14 | 0.82 |
| **PAR** | -0.83 | 0.08 | 0.31 | 0.61 | -0.52 | 0.36 | 0.76 | 0.13 |
| **Temperature** | -0.89 | *0.05 | 0.31 | 0.62 | -0.96 | **0.01 | 0.24 | 0.70 |
| **Ammonium** | 0.96 | *0.01 | 0.02 | 0.97 | 0.68 | 0.20 | -0.55 | 0.33 |
| **Nitrate** | 0.84 | 0.07 | 0.23 | 0.72 | 0.31 | 0.62 | -0.70 | 0.19 |
| **Silicate** | -0.61 | 0.28 | 0.07 | 0.91 | -0.90 | *0.04 | -0.33 | 0.59 |
| **Phosphate** | 0.93 | *0.02 | 0.16 | 0.80 | 0.46 | 0.43 | -0.68 | 0.21 |

**S6-** α-diversity of the communities recovered from each station. Observed OTU richness is the number of OTUs recovered. Estimated OTUs is the OTU richness predicted by each corresponding method.

|  | | **Group** | | **Observed OTU richness** | | | | **Estimated OTU richness (iNEXT)** | | | | **Estimated OTU richness (ACE)** | | | | **Shannon diversity** | | | | **Simpson diversity** | | | |
| --- | --- | --- | --- | --- | --- | --- | --- | --- | --- | --- | --- | --- | --- | --- | --- | --- | --- | --- | --- | --- | --- | --- | --- |
|  | |  | | Eukaryotes | | Bacteria | | Eukaryotes | | Bacteria | | Eukaryotes | | Bacteria | | Eukaryotes | | Bacteria | | Eukaryotes | | Bacteria | |
| **CTD56** | | LI | | 983 | | 4 556 | | 1 113 | | 6 684 | | 1 115 | | 6 949 | | 38.7 | | 464.2 | | 13.1 | | 83.3 | |
| **CTD57** | | LI | | 851 | | 4 094 | | 1 039 | | 5 750 | | 1 065 | | 5 969 | | 21.7 | | 384.4 | | 9.7 | | 68.5 | |
| **CTD58** | | MI | | 1 344 | | 4 482 | | 1 434 | | 7 095 | | 1 482 | | 7 449 | | 76.9 | | 433.2 | | 22.8 | | 65.8 | |
| **CTD59** | | HI | | 963 | | 4 324 | | 1 263 | | 7 708 | | 1 317 | | 8 025 | | 26.3 | | 308.2 | | 10.2 | | 39.4 | |
| **CTD62** | | HI | | 940 | | 3 630 | | 1 043 | | 5 772 | | 1 042 | | 5 991 | | 28.4 | | 248.4 | | 9.2 | | 41.4 | |
| **Total** | |  | | 2 558 | | 10 272 | | 2 739 | | 14 300 | | 2826 | | 14620 | |  | |  | |  | |  | |
|  | |  | |  | |  | |  | |  | |  | |  | |  | |  | |  | |  | |
| **CTD08** | | NI | | 840 | | 4 279 | | 1 024 | | 6 856 | | 1 029 | | 7 218 | | 10.8 | | 359.3 | | 4.0 | | 57.8 | |
| **CTD10** | | NI | | 1 201 | | 2 979 | | 1 411 | | 4 433 | | 1 425 | | 4 458 | | 30.3 | | 225.5 | | 11.2 | | 48.5 | |
| **CTD12** | | NI | | 1 370 | | 3 430 | | 1 685 | | 4 647 | | 1 731 | | 4 940 | | 51.2 | | 267.0 | | 17.5 | | 45.7 | |

**S7- Bacterial and Eukaryotic community composition.** Shown is the relative abundance of each of the constituent major taxonomic groups for the a) bacterial and b) eukaryotic community.

**
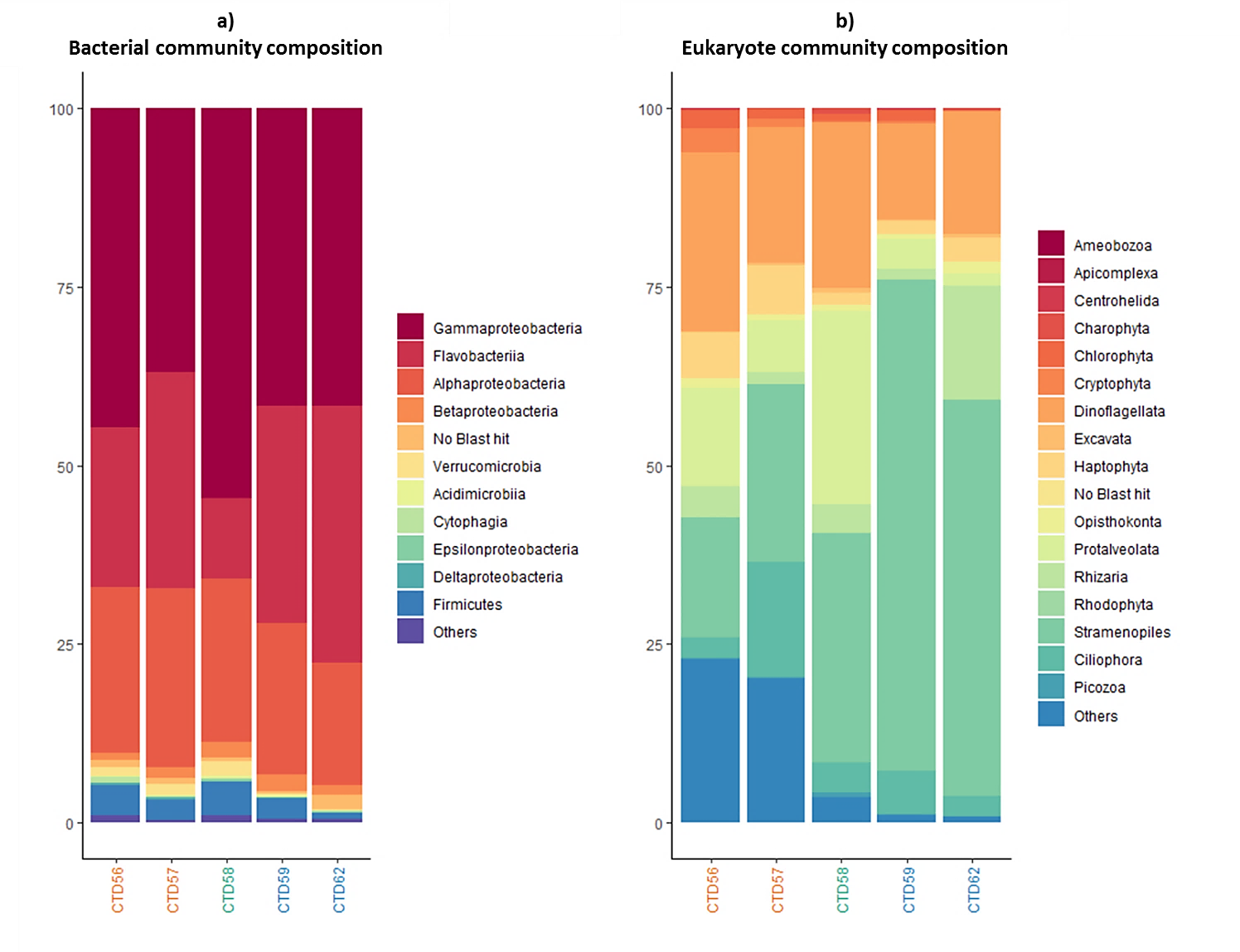
**

**S8- Dendrogram of the Bray-Curtis dissimilarity matrix of OTUs between stations.** Shown is the partitioning of the a) eukaryotic community including copepods, to highlight the affect potential mesozooplankton debris had upon the analysis. Removal of these OTUs resulted in a reduced dissimilarity of HI stations to one another. Also show is the partitioning of the b) eukaryotes and c) bacteria including stations collected in the North Atlantic for the validation of the observed grouping. Stations are coloured based upon the extent of Polar Water influence determined to be present at each station as in Figure 1; orange- LI, green- MI, blue- HI, pink – North Atlantic stations.

**
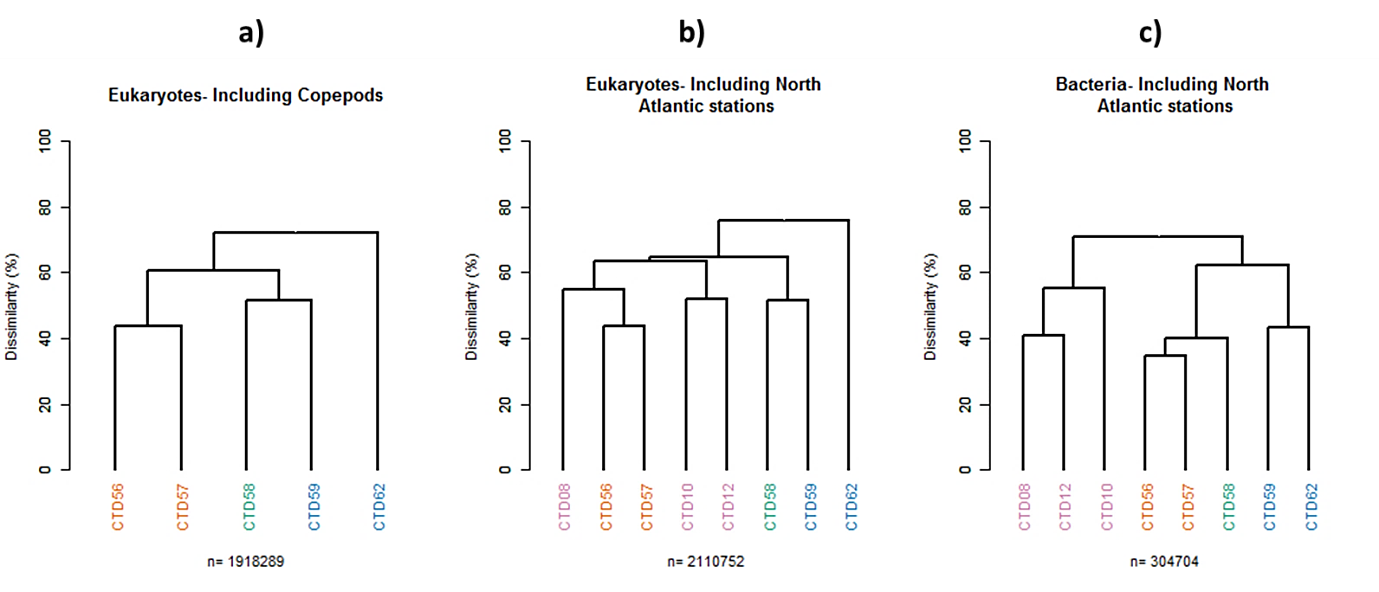
**

**S9- The proportional contribution of selected bacterial OTUs to the community across all stations.** OTUs were selected from the top 200 most abundant for the bacterial dataset. Shown is the proportional contribution each OTU makes to the community across all stations. OTUs show different distribution patterns which help explain the community assemblage structure and can be grouped into those which are present across all stations (a), proportionally higher in LI stations (b), proportionally higher in the MI station (c), or are proportionally higher within the HI stations (d). Stations are coloured based upon the degree of Polar Water influence determined to be present at each station; orange- LI, green- MI, blue- HI. ** indicates a significant correlation with temperature. * indicates a significant correlation with other environmental variables.


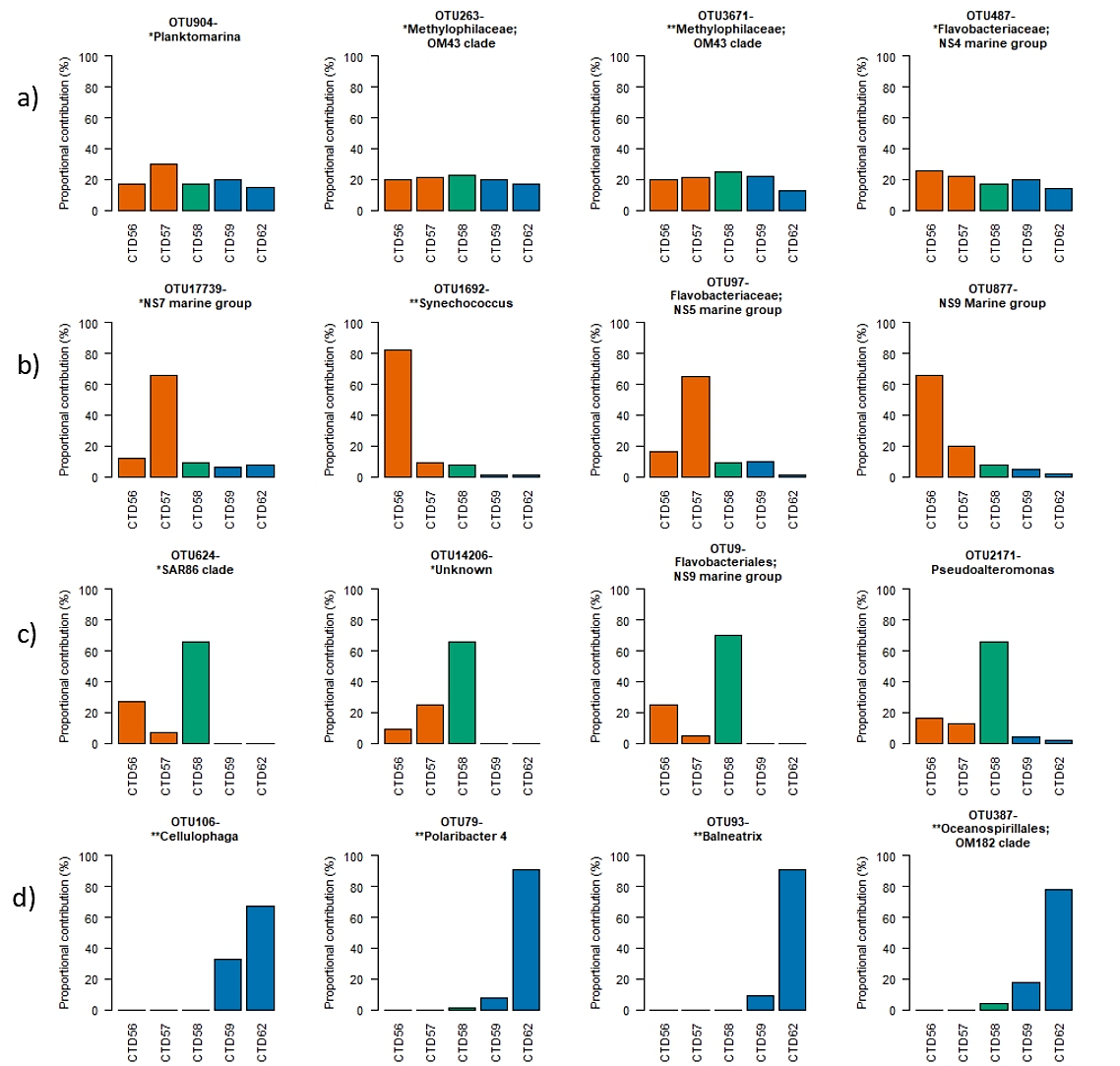


**S10- The proportional contribution of selected Eukaryotic OTUs to the community across all stations.** OTUs were selected from the top 200 most abundant for the Eukaryotic dataset. Shown is the proportional contribution each OTU makes to the community across all stations. OTUs show different distribution patterns which help explain the community assemblage structure and can be grouped into those which are present across all stations (a), proportionally higher in LI stations (b), proportionally higher in the MI station (c), or are proportionally higher within the HI stations (d). Stations are coloured based upon the degree of Polar Water influence determined to be present at each station; orange- LI, green- MI, blue- HI. ** indicates a significant correlation with temperature. * indicates a significant correlation with other environmental variables.


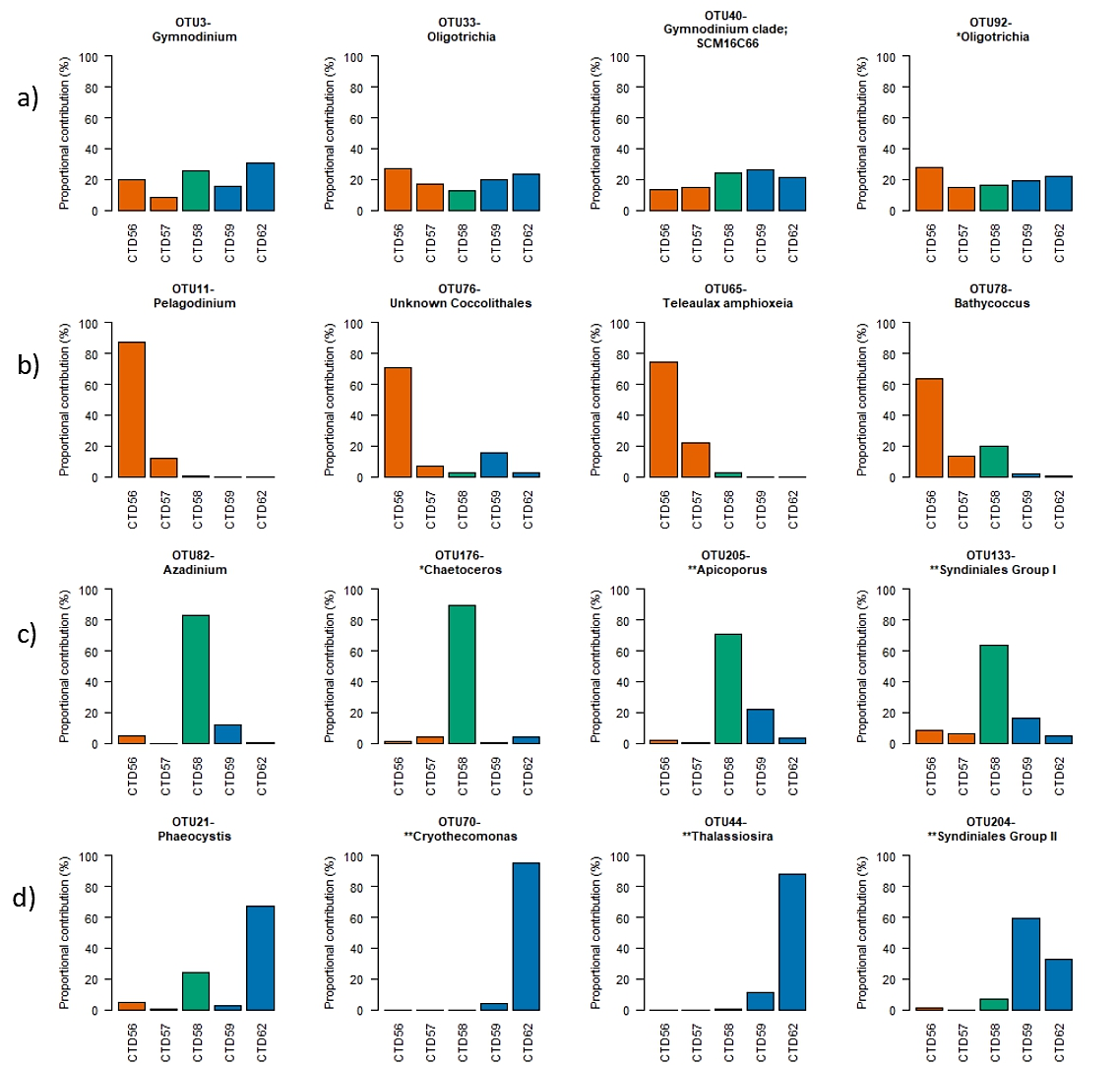


**S11- The proportional contribution of selected bacterial OTUs, that displayed more graduated distribution patterns, to the community across all stations.** OTUs were selected from the top 200 most abundant for the bacterial dataset. Shown is the proportional contribution each OTU makes to the community across all stations. OTUs show more graduated distribution patterns which help explain the community assemblage structure, but can still be seen to broadly reflect those which are proportionally higher in LI stations (a), proportionally higher in the MI station (b), or are proportionally higher within the HI stations (c). Stations are coloured based upon the degree of Polar Water influence determined to be present at each station; orange - LI, green- MI, blue- HI. ** indicates a significant correlation with temperature. * indicates a significant correlation with other environmental variables.


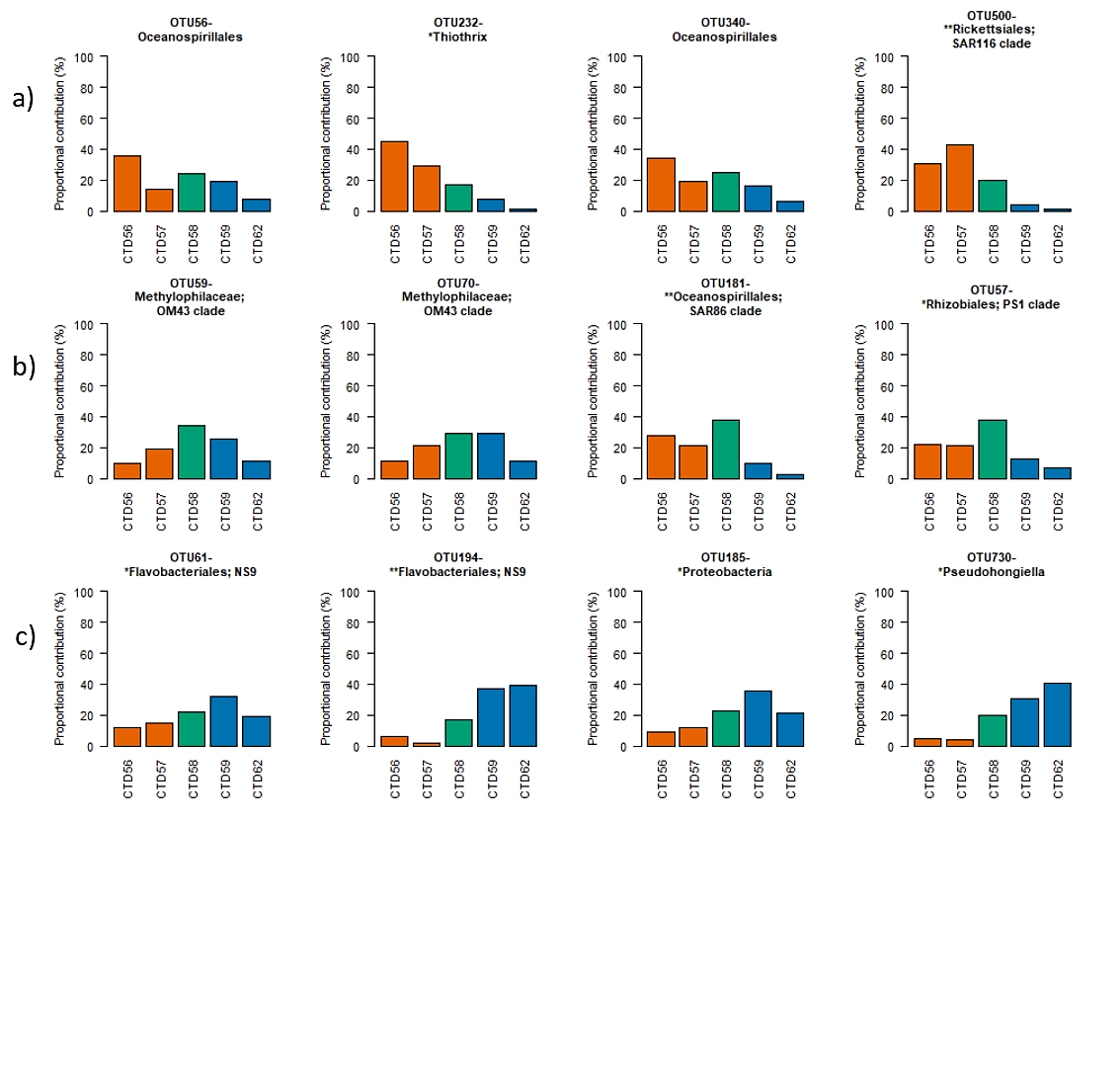


**S12 -** The CTD casts from which environmental samples were taken aboard the RRS James Clarke Ross research Vessel during the JR271 cruise (1st June 2012 to 2nd July 2012).

| **Station number** | **CTD cast** | **Original collection area** | **Latitude (DD.dddddd°)** | **Longitude (DD.dddddd°)** |
| --- | --- | --- | --- | --- |
| 24 | JR271 CTD 08 | *North and North-West of Scotland (North Atlantic Ocean)* | 60.1342 | -6.7121 |
| 25 | JR271 CTD 10 | *North and North-West of Scotland (North Atlantic Ocean)* | 59.9710 | -11.9751 |
| 23 | JR271 CTD 12 | *South West of Iceland (North Atlantic Ocean)* | 60.0014 | -18.6702 |
| 12 | JR271 CTD 56 | *Norwegian Sea* | 71.7475 | 8.4428 |
| 13 | JR271 CTD 57 | *Norwegian Sea* | 71.7519 | 3.8717 |
| 14 | JR271 CTD 58 | *Norwegian Sea* | 71.7453 | -1.2672 |
| 15 | JR271 CTD 59 | *Norwegian Sea* | 71.7517 | -5.8638 |
| 16 | JR271 CTD 62 | *Norwegian Sea* | 70.5083 | -10.1000 |

**S13-** The nucleotide sequences for each of the forward and reverse primers used during the probe assay. Full descriptions of each primer are displayed and delineated by ‘/’. Target region is the region of the 18S or 16S region to be amplified. Adapter is the adaptor sequence required to immobilise the sequence for amplification on the Illumina flow cell. Primer pad is a region to avoid primer-dimer formation. Primer linker is a sequence to prevent taxon specific PCR bias. Primer is the complimentary sequence to the target DNA barcode.

| **Target region** | **Primer name** | **Nucleotide sequence (Illumina adapter / primer pad / primer linker / primer)** |
| --- | --- | --- |
| 18S V9 | 1391F | AATGATACGGCGACCACCGAGATCTACAC / TATGGTAATT / GT / GTACACACCGCCCGTC |
| 18S V9 | EukB 2 | CAAGCAGAAGACGGCATACGAGAT / AGGACGCACTGT / AGTCAGTCAG / CC / TGATCCTTCTGCAGGTTCACCTAC |
| 18S V9 | EukB 9 | CAAGCAGAAGACGGCATACGAGAT / ACAGAGTCGGCT / AGTCAGTCAG / CC / TGATCCTTCTGCAGGTTCACCTAC |
| 18S V9 | EukB 4 | CAAGCAGAAGACGGCATACGAGAT / AACTCGTCGATG / AGTCAGTCAG / CC / TGATCCTTCTGCAGGTTCACCTAC |
| 18S V9 | EukB 11 | CAAGCAGAAGACGGCATACGAGAT / ACGGTGAGTGTC / AGTCAGTCAG / CC / TGATCCTTCTGCAGGTTCACCTAC |
| 18S V9 | EukB 12 | CAAGCAGAAGACGGCATACGAGAT / ACTCGATTCGAT / AGTCAGTCAG / CC / TGATCCTTCTGCAGGTTCACCTAC |
| 18S V9 | EukB 13 | CAAGCAGAAGACGGCATACGAGAT / AGACTGCGTACT / AGTCAGTCAG / CC / TGATCCTTCTGCAGGTTCACCTAC |
| 18S V9 | EukB 14 | CAAGCAGAAGACGGCATACGAGAT / AGCAGTCGCGAT / AGTCAGTCAG / CC / TGATCCTTCTGCAGGTTCACCTAC |
| 18S V9 | EukB 20 | CAAGCAGAAGACGGCATACGAGAT / AGACGTGCACTG / AGTCAGTCAG / CC / TGATCCTTCTGCAGGTTCACCTAC |
| 16S V4-V5 | 515F | AATGATACGGCGACCACCGAGATCTACAC / TATGGTAATT / GT / GTGCCAGCMGCCGCGGTAA |
| 16S V4-V5 | 806R 1 | CAAGCAGAAGACGGCATACGAGAT / AACGCACGCTAG / AGTCAGTCAG / CC / GGACTACHVGGGTWTCTAAT |
| 16S V4-V5 | 806R 4 | CAAGCAGAAGACGGCATACGAGAT / ACTCAGATACTC / AGTCAGTCAG / CC / GGACTACHVGGGTWTCTAAT |
| 16S V4-V5 | 806R 5 | CAAGCAGAAGACGGCATACGAGAT / ACCAGACGATGC / AGTCAGTCAG / CC / GGACTACHVGGGTWTCTAAT |
| 16S V4-V5 | 806R 9 | CAAGCAGAAGACGGCATACGAGAT / ACGGATCGTCAG / AGTCAGTCAG / CC / GGACTACHVGGGTWTCTAAT |
| 16S V4-V5 | 806R 10 | CAAGCAGAAGACGGCATACGAGAT / AGCTGACTAGTC / AGTCAGTCAG / CC / GGACTACHVGGGTWTCTAAT |
| 16S V4-V5 | 806R 11 | CAAGCAGAAGACGGCATACGAGAT / ACACTGTTCATG / AGTCAGTCAG / CC / GGACTACHVGGGTWTCTAAT |
| 16S V4-V5 | 806R 13 | CAAGCAGAAGACGGCATACGAGAT / ACAGACCACTCA / AGTCAGTCAG / CC / GGACTACHVGGGTWTCTAAT |
| 16S V4-V5 | 806R 15 | CAAGCAGAAGACGGCATACGAGAT / ACCAGCGACTAG / AGTCAGTCAG / CC / GGACTACHVGGGTWTCTAAT |

**S14-** The primers used and quantity of DNA extracted for probe assays. Forward and reverse primers feature different barcode sequences, not primer sequences to ensure the same DNA region is amplified.

| **Sample** | **16s Forward Primer** | **16s Reverse Primer** | **DNA (ng/µl)** | **Sample** | **18s Forward Primer** | **18s Reverse Primer** | **DNA (ng/µl)** |  |
| --- | --- | --- | --- | --- | --- | --- | --- | --- |
| CTD 8 16S REP 1 | 515F | 806R 1 | 3.40 | CTD 8 18S REP 1 | 1391F | EukB 2 | 8.88 |  |
| CTD 8 16S REP 2 | 515F | 806R 1 | 21.09 | CTD 8 18S REP 2 | 1391F | EukB 2 | 18.86 | |
| CTD 8 16S REP 3 | 515F | 806R 1 | 20.27 | CTD 8 18S REP 3 | 1391F | EukB 2 | 26.98 |  |
| CTD 8 16S REP 4 | 515F | 806R 1 | 15.06 |  |  |  |  |  |
| CTD 10 16S REP 1 | 515F | 806R 4 | 8.71 | CTD 10 18S REP 1 | 1391F | EukB 9 | 11.92 |  |
| CTD 10 16S REP 2 | 515F | 806R 4 | 7.69 | CTD 10 18S REP 2 | 1391F | EukB 9 | 26.06 |  |
| CTD 10 16S REP 3 | 515F | 806R 4 | 5.23 | CTD 10 18S REP 3 | 1391F | EukB 9 | 25.72 |  |
| CTD 12 16S REP 1 | 515F | 806R 5 | 3.10 | CTD 12 18S REP 1 | 1391F | EukB 4 | 4.95 |  |
| CTD 12 16S REP 2 | 515F | 806R 5 | 11.31 | CTD 12 18S REP 2 | 1391F | EukB 4 | 10.45 |  |
| CTD 12 16S REP 3 | 515F | 806R 5 | 18.21 | CTD 12 18S REP 3 | 1391F | EukB 4 | 6.27 |  |
| CTD 12 16S REP 4 | 515F | 806R 5 | 5.98 |  |  |  |  |  |
| CTD 56 16S REP 1 | 515F | 806R 9 | 5.56 | CTD 56 18S REP 1 | 1391F | EukB 11 | 7.25 |  |
| CTD 56 16S REP 2 | 515F | 806R 9 | 24.71 | CTD 56 18S REP 2 | 1391F | EukB 11 | 10.62 |  |
| CTD 56 16S REP 3 | 515F | 806R 9 | 13.04 | CTD 56 18S REP 3 | 1391F | EukB 11 | 22.14 |  |
| CTD 57 16S REP 1 | 515F | 806R 10 | 6.04 | CTD 57 18S REP 1 | 1391F | EukB 12 | 18.70 |  |
| CTD 57 16S REP 2 | 515F | 806R 10 | 16.97 | CTD 57 18S REP 2 | 1391F | EukB 12 | 14.26 |  |
| CTD 57 16S REP 3 | 515F | 806R 10 | 29.64 | CTD 57 18S REP 3 | 1391F | EukB 12 | 20.26 |  |
| CTD 58 16S REP 1 | 515F | 806R 11 | 4.45 | CTD 58 18S REP 1 | 1391F | EukB 13 | 9.45 |  |
| CTD 58 16S REP 2 | 515F | 806R 11 | 15.93 | CTD 58 18S REP 2 | 1391F | EukB 13 | 9.76 |  |
| CTD 58 16S REP 3 | 515F | 806R 11 | 15.17 | CTD 58 18S REP 3 | 1391F | EukB 13 | 19.26 |  |
| CTD 58 16S REP 4 | 515F | 806R 11 | 26.09 |  |  |  |  |  |
| CTD 59 16S REP 1 | 515F | 806R 13 | 5.71 | CTD 59 18S REP 1 | 1391F | EukB 14 | 5.77 |  |
| CTD 59 16S REP 2 | 515F | 806R 13 | 3.98 | CTD 59 18S REP 2 | 1391F | EukB 14 | 9.49 |  |
| CTD 59 16S REP 3 | 515F | 806R 13 | 10.34 | CTD 59 18S REP 3 | 1391F | EukB 14 | 13.33 |  |
| CTD 62 16S REP 1 | 515F | 806R 15 | 13.68 | CTD 62 18S REP 1 | 1391F | EukB 20 | 8.00 |  |
| CTD 62 16S REP 2 | 515F | 806R 15 | 9.49 | CTD 62 18S REP 2 | 1391F | EukB 20 | 12.69 |  |
| CTD 62 16S REP 3 | 515F | 806R 15 | 3.59 | CTD 62 18S REP 3 | 1391F | EukB 20 | 13.44 |  |

**S15-** Rarefaction (solid line) and extrapolation (dashed line) of OTU richness for the a) rarefied eukaryotic, and b) bacterial dataset. Legend displays each station for which rarefaction was calculated as well as the % saturation of the recovered OTU number of the total number of estimated OTUs with standard error.


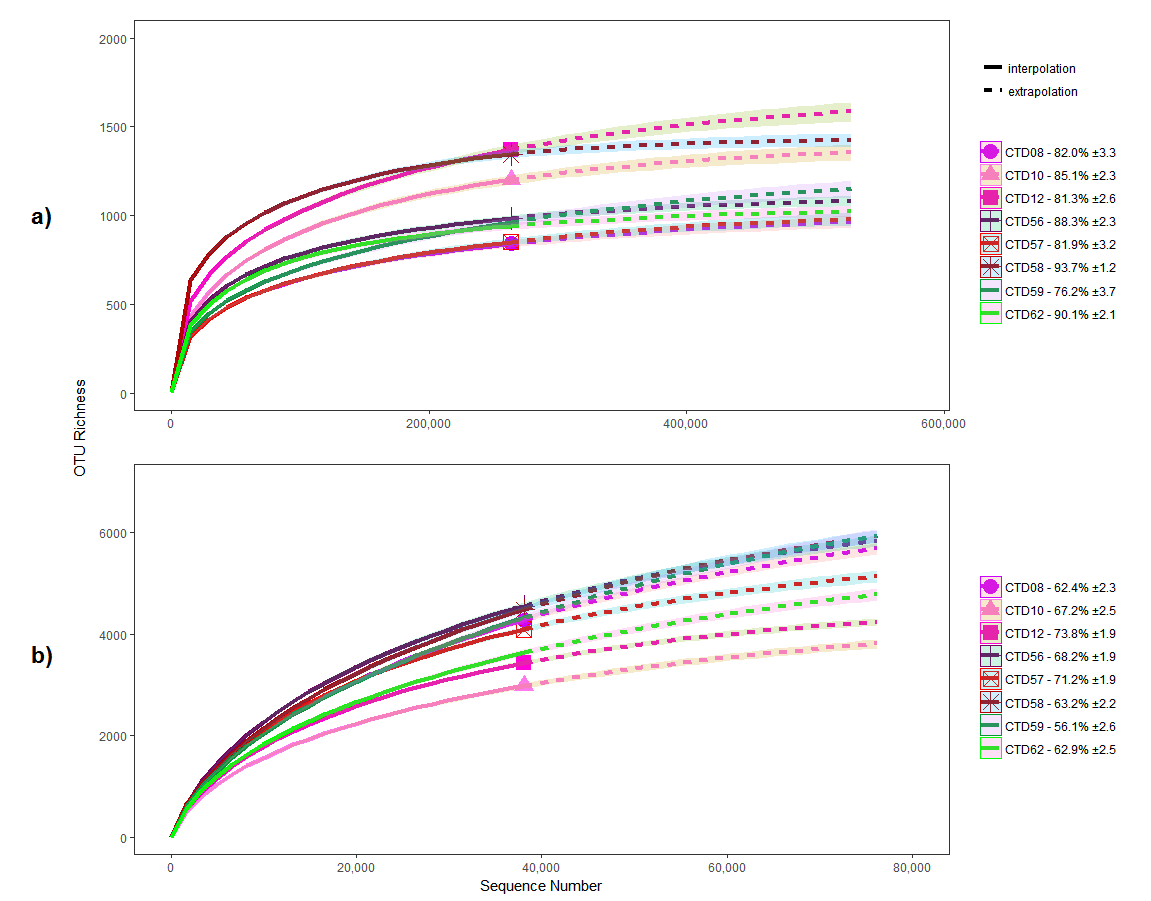

Supplement: Supplementary file 1 — Supplementary Information. [file 41598_2020_76293_MOESM1_ESM.docx]
